# Rates of recruitment from systematic and opportunistic methods: preliminary results from the DDELPHI study

**DOI:** 10.1186/1745-6215-12-S1-A112

**Published:** 2011-12-13

**Authors:** Melvyn Hillsdon

**Affiliations:** 1 University of Exeter, Sport and Health Sciences, St Luke's Campus, Heavitree Road, Exeter, Devon, EX16 7NW, UK

## Background

Problems with recruitment at both practice and patient level frequently lead to low response rates in primary care trials that can compromise study results. As part of a trial feasibility study of the effectiveness of brief advice from general practitioners to encourage daily walking, we examined the association between practice characteristics and the recruitment of practices and patients. We also examined differences in recruitment rates between two methods of patient recruitment.

## Methods

Practices in Coventry, Bristol and Devon were invited to take part in the study (n=114) via letter and asked to indicate an expression of interest (EOI) for participation. Of the 37 interested practices, 24 were randomly assigned to cluster randomisation (n=18) or individual patient randomisation (n=6) and then randomised to recruit patients either opportunistically via practice waiting rooms or systematically by post from patient lists. One hundred and forty four patients were recruited. Practice list sizes were categorised as low (<3,500), medium (3,500-8,000) and large (>8,000). Using the home postcodes of patients and the English Index of Multiple Deprivation, practice deprivation levels were categorised as low (lowest quartile of IMD in England) middling (inter-quartile range) and high (highest quartile of IMD).

## Results

It took an average of 22.6 days (SD 14.7) for practices to give an EOI in Devon, 31.5 days (SD 13.3) in Bristol and 44.3 days (SD 20.4) in Coventry. Time to EOI was 40.7 (SD 22.2), 26.9 (17.2) and 29.6 (SD 13.6) days for small, medium and large practices respectively and 25.0 (SD 2.6), 33.6 (SD 20.0) and 31.2 days for low, middling and high deprivation practices. Devon practices averaged 163.6 (SD 33.5) days to recruit the first patient after EOI compared to 123.5 (SD 35.2) days in Bristol and 134.6 (SD 32.7) days in Coventry. Time from EOI to recruitment of first patient was unrelated to practice size but low deprivation practices averaged 19.4 days less than middling practices and 17.2 days less than high deprivation practices to recruit the first patient. Opportunistic recruitment took an average of 132.3 (SD 35.0) days to recruit compared to 151.3 (SD 37.0) days for patients recruited by letter.

## Conclusions

There are important differences in time taken to recruit both practices and/or patients according to geographical location of the practice, practice list size and deprivation score as well as the method of patient recruitment.

